# Setup errors and effectiveness of Optical Laser 3D Surface imaging system (Sentinel) in postoperative radiotherapy of breast cancer

**DOI:** 10.1038/s41598-018-25644-w

**Published:** 2018-05-08

**Authors:** Xiaobo Wei, Mengjiao Liu, Yun Ding, Qilin Li, Changhai Cheng, Xian Zong, Wenming Yin, Jie Chen, Wendong Gu

**Affiliations:** Department of Radiation Oncology, The Third Affiliated Hospital of Soochow University, 185 Juqian Street, Changzhou, 213003 People’s Republic of China

## Abstract

Breast-conserving surgery (BCS) plus postoperative radiotherapy has become the standard treatment for early-stage breast cancer. The aim of this study was to compare the setup accuracy of optical surface imaging by the Sentinel system with cone-beam computerized tomography (CBCT) imaging currently used in our clinic for patients received BCS. Two optical surface scans were acquired before and immediately after couch movement correction. The correlation between the setup errors as determined by the initial optical surface scan and CBCT was analyzed. The deviation of the second optical surface scan from the reference planning CT was considered an estimate for the residual errors for the new method for patient setup correction. The consequences in terms for necessary planning target volume (PTV) margins for treatment sessions without setup correction applied. We analyzed 145 scans in 27 patients treated for early stage breast cancer. The setup errors of skin marker based patient alignment by optical surface scan and CBCT were correlated, and the residual setup errors as determined by the optical surface scan after couch movement correction were reduced. Optical surface imaging provides a convenient method for improving the setup accuracy for breast cancer patient without unnecessary imaging dose.

## Introduction

Radiotherapy after early-stage breast-conserving surgery (BCS) can increase the 5-year local control by 19%, and the 15-year overall survival benefit added is about 5%^1^. Modern treatment techniques such as intensity modulated radiotherapy (IMRT) and volumetric modulated arc radiotherapy (VMAT) can further improve the dosimetry in the target and surrounding healthy tissues in terms of target dose conformity and uniformity, and reduced skin dose. Delivery of highly conformal dose distribution requires accurate patient setup^2^. Image-guided radiotherapy (IGRT) using electronic portal imaging detector (EPID) or cone-beam computerized tomography (CBCT) can help reduce patient setup errors but inevitably increases the setup time with burdens of added work flow for positioning correction and extra imaging dose to patients^3^. Three dimensional (3D) optical surface imaging is a fast and non-invasive method for assisting patient setup, which is particularly suitable for patients with breast cancer^4^. The purpose of this study is to compare the setup accuracy of optical surface imaging by the Sentinel system with CBCT imaging currently used in our clinic for BCS patients.

## Results

A total of 145 sets of CE, SE-before and SE-after scans in 27 patients were analyzed. The numbers of treatment sessions for individual patients were from 4 to 7, and the median was 5.

**Figure 1:**
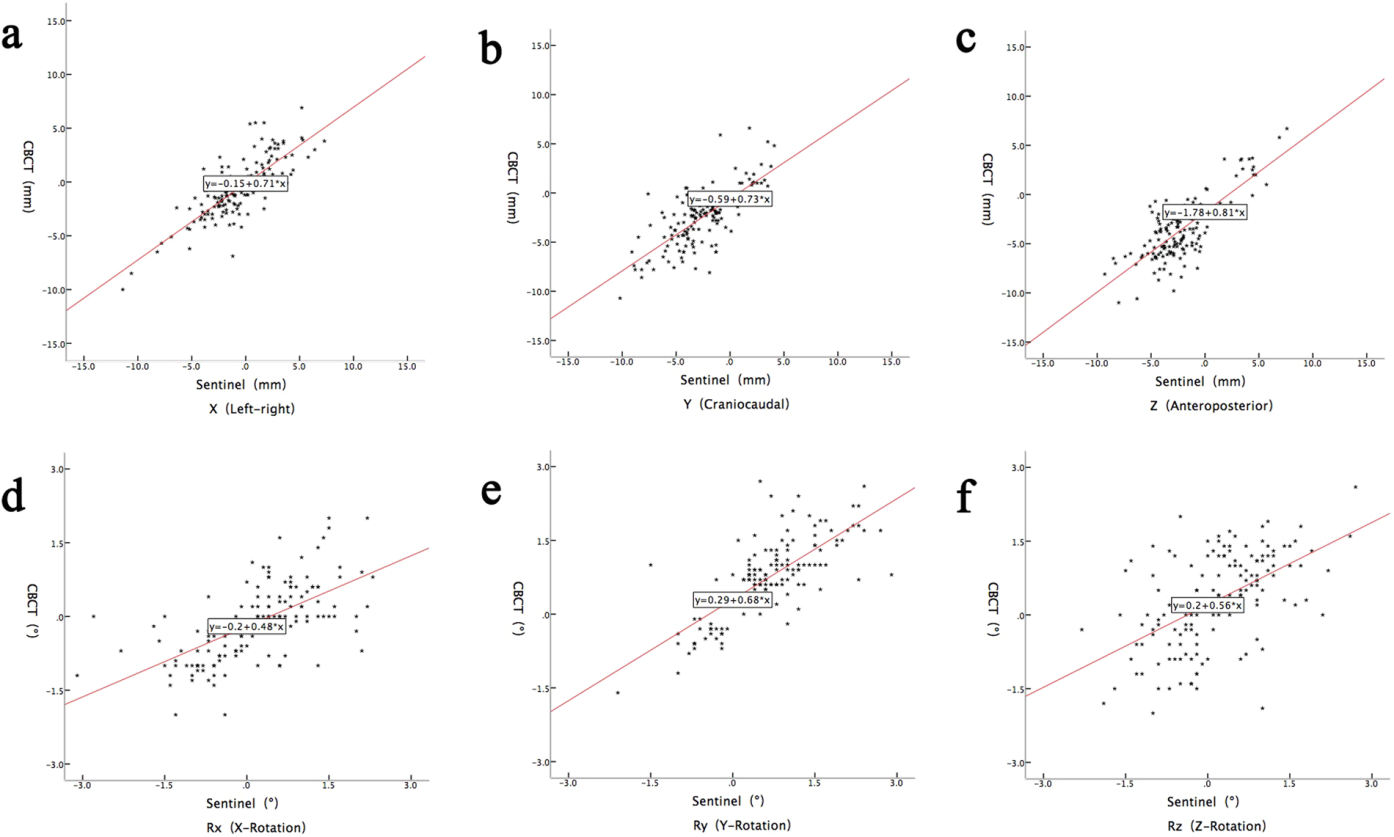
The scatter spot of CBCT and Sentinel error before moving couch. The correlation of CE and SE-before in the three translation directions (**a**–**c**). The correlation of CE and SE-before in the three rotations about the X, Y, Z axes (**d**–**f**).

The correlation coefficient r of CE and SE-before errors in every dimension were between 0.541~0.796, indicating significant correlations, and the p-values were less than 0.001 (Table 1 and Fig. 1).

**Table 1:** Correlation studies of CE and SE-before.

	CE	SE-before	Correlation Coefficient	P-value
X(left-right)	8 mm ± 2.79 mm	−0.62 mm ± 3.13 mm	0.796	<0.001
Y(cranial-caudal)	−2.55 mm ± 2.97 mm	−2.67 mm ± 2.93 mm	0.723	<0.001
Z(anteroposterior)	−3.58 mm ± 3.17 mm	−2.21 mm ± 2.97 mm	0.765	<0.001
Rx(x-rotation)	−0.1° ± 0.8°	0.2° ± 1.0°	0.63	<0.001
Ry(y-rotation)	0.7° ± 0.8°	0.6° ± 0.9°	0.741	<0.001
Rz(z-rotation)	0.3° ± 1.0°	0.2° ± 0.9°	0.541	<0.001

CE: cone beam CT errors; SE-before: sentinel errors before the treatment couch correction.

Table 2 shows the systemic error and random errors for CE and SE-before. These errors were comparable between the two alignment methods with the differences in X, Y, and Z directions by 0.13 mm and 0.53 mm, 0.28 mm and 0.34 mm, and 0.34 mm and 0.17 mm, respectively. The rotational errors were also very similar for the two methods with the differences less than 0.2°. Accordingly, the anisotropic PTV margins derived from Sentinel were different from the CBCT data by 0.63 mm, 0.33 mm, and 0.56 mm in X, Y, and Z directions, respectively. The impact of rotational errors to the PTV margins are expected to be included.

**Table 2:** Setup error of CBCT and sentinel before moving the treatment table.

		The group mean (M)	Systemic error (Σ)	Random error (δ)	PTV margin
X-shift	CBCT (mm)	−0.64	2.49	1.45	6.00
	Sentinel (mm)	−0.67	2.62	1.98	6.63
Y-shift	CBCT (mm)	−2.47	2.80	1.48	6.64
	Sentinel (mm)	−2.64	2.52	1.82	6.31
Z-shift	CBCT (mm)	−3.59	3.04	1.51	7.14
	Sentinel (mm)	−2.19	2.70	1.68	6.58
X-rotation	CBCT (°)	− 0.1	0.6	0.5	—
	Sentinel (°)	0.1	0.8	0.7	—
Y-rotation	CBCT (°)	0.7	0.7	0.5	—
	Sentinel (°)	0.6	0.6	0.6	—
Z-rotation	CBCT (°)	0.3	0.8	0.7	—
	Sentinel (°)	0.2	0.6	0.8	—

CBCT: cone-beam computerized tomography.

The residual errors based on the Sentinel surface imaging scanned after the couch movement are given in Table 3. The PTV margins derived from the data were reduced to 4.49 mm, 4.85 mm, 3.67 mm in X, Y, and Z directions, respectively.

**Table 3:** Residual setup error of Sentinel.

	The group mean (M)	Systemic errors (∑)	Random errors (σ)	PTV margin
X-shift (mm)	0.1	1.7	1.56	4.49
Y-shift (mm)	1.36	1.85	1.64	4.85
Z-shift (mm)	−1.28	1.32	1.47	3.67
X-rotation (°)	0.3	0.6	0.7	—
Y-rotation (°)	−0.1	0.9	0.7	—
Z-rotation (°)	0.1	0.7	0.8	—

PTV: planning target volume.

## Discussion

BCS plus postoperative radiotherapy has become the standard treatment for early-stage breast cancer. Treatment techniques for BCS have been improved in recent couple of decades, aiming at minimizing the dose to the lung and heart. The use of IMRT not only increases the target dose uniformity but also reduces the dose to surrounding normal tissues, especially the heart and coronary arteries for the left side treatment^5^. However, the changes in breast shape and size due to variations of patients’ position and tissue inflammatory reaction such as postoperative serum like swelling, along with respiratory motion, may lead to dose delivery errors^6^. As result the benefit in local control and side effect reduction of IMRT may get nullified^7^. However, accurate patient positioning could increase the dose coverage to the CTV by 10% while significantly reduced the exposure to the lung and heart^8^. This means that patient setup accuracy, specifically the positioning of the target volume position, is important for the delivered dose distribution^9^. Therefore, to ensure the accuracy and reproducibility of the patient positioning is paramount to the safe and effective implementation of radiation therapy for BCS. The use of EPID, CBCT and other image-guided equipment can detect and correct the setup error either offline or online, which greatly improves the accuracy of treatment^10^. However, X-ray based IGRT process involves additional radiation to patients^11^. Studies showed that the dose of the single IGRT was about 0.01 mGy~1 mGy, for example, see^12^. The imaging dose of EPID was the highest, followed by KV- CBCT, and KV portal film the lowest. The dose with EPID was almost 2~10 times bigger than that of CBCT^13^. Surface imaging techniques can obtain the patients’ contour in three-dimensions with millimeter accuracy, which can be used to assist patient setup conveniently without any radiation.

Semaniak *et al*.^14^ measured the setup errors in two groups of 130 patients with radiotherapy after modified radical mastectomy by using EPID. It was found that for the left-right direction Σ were 1.6 mm and 1.7 mm, and δ were 1.6 mm and 1.3 mm, respectively. The resulted PTV margin were 5.1 mm and 5.4 mm for these two groups of patients, respectively. In cranial-caudal direction Σ were 1.5 mm and 1.9 mm, δ were 1.7 mm and 1.3 mm, respectively. The cranial-caudal PTV margins were 4.9 mm and 6.4 mm, respectively. Chung *et al*.^15^ studied the setup errors of 147 patients with postoperative radiotherapy for early-stage breast cancer by using MVCT (Megavoltage Computerized Tomography). They found that Σ and δ were 1.98 mm and 1.87 mm in left-right, 2.02 mm and 2.10 mm in cranial-caudal, and 2.99 mm and 2.82 mm in anterior-posterior direction, respectively. According to the Stroom formula^16^, the PTV margins were 5.27 mm, 5.51 mm and 7.95 mm respectively. In our study, PTV margins based on CBCT were 6.00 mm, 6.64 mm and 7.14 mm in left-right, cranial-caudal, and anterior-posterior direction, and this was similar to the above two studies. Another study by Lakosi *et al*.^17^ used CBCT to study the setup errors of 36 cases of breast cancer in the prone position. Their results showed that Σ and δ were about 4.5 mm and 5.4 mm in left-right, 3.9 mm and 3.8 mm in cranial-caudal, 3.3 mm and 2.8 mm in anterior-posterior direction, the PTV margins were 15.0 mm, 12.3 mm and 10.3 mm, respectively. The setup errors were significantly greater than that of Semaniak *et al*.^14^, Chung *et al*.^15^ and our study. This means that prone treatment could involve larger uncertainties than that in supine treatment.

Walter *et al*.^18^ retrospectively analyzed a total of 154 surface imaging (Catalyst) scans in 25 patients with thoracic, abdominal and pelvic tumors. They found that the setup errors of CBCT were 0.0 ± 2.1 mm in left-right, −0.4 ± 2.4 mm in cranial-caudal, and 1.1 ± 2.6 mm in anterior-posterior direction; and the setup errors by Catalyst scan in the three translation directions were −0.1 ± 2.1 mm, −1.8 ± 5.4 mm and 1.4 ± 3.2 mm, respectively. There was no significant difference between the two approaches, which led the authors to believe that Catalyst could be used as at least an effective alternative to CBCT. Crop *et al*.^19^ studied the setup error based on laser light, Catalyst and mega voltage computed tomography (MVCT) in postoperative radiotherapy for breast cancer. The results showed that the setup error of the Catalyst was significantly better than that of laser-based positioning and was very close to the MVCT. However, Padilla *et al*.^20^ reported negative results in their study of the correlation of surface imaging (AlignRT) and MV portal imaging in postoperative radiotherapy for breast cancer. The AlignRT showed poor correlation with MV portal imaging in cranial-caudal direction (r = 0.14), and only moderate correlations (r = 0.49 and 0.66) in anterior-posterior and left-right direction. Our results showed a significant correlation between CBCT and Sentinel errors (r = 0.541–0.796) and statistical significance with p-values less than 0.001. The differences in setup errors between CBCT and Sentinel, in terms of Σ, δ, and PTV margins, were less than 1 mm and 0.5°. Our result was similar to Walter *et al*.^18^ and Crop *et al*.^19^, but significantly better than Padilla *et al*.^20^. One can argue that using MV images did not take the shape of the breast and deformation in the treatment process into account, while Sentinel and other surface imaging techniques is sensitive to changes in shape and size of breast^21^. In our study the ROI of Sentinel scan only included the treated breast but excluded the chin, the ipsilateral upper limb, and armpit, which were likely to have an impact on matching. Likewise, the clip box for CBCT matching was limited to the region of treated breast and surrounding tissues. Manual adjustment performed after auto-registration could have improved the accuracy. A study to compare the accuracy of EPID and CBCT in BCS found that using MV images underestimated the setup errors by 20% to 50%^22^.

The Sentinel scan uses patient’s surface in 3D to match with that reconstructed from planning CT, instead of bony markers distant from the target volume. This makes it especially suitable for breast cancer treatment with the target volume situated close to the body surface. Batin *et al*.^21^ used radioopaque metal markers placed on the skin near the tumor bed to replace the bony marker away from the target volume, in order to improve the accuracy of portal imaging assisted setup. The results with portal imaging were unsatisfactory due to the limited area that the metal marker surrogates could identify. Furthermore, there was an uncertainty in placing those metal markers reproducibly for each treatment. On the contrary, the reference for matching with optical surface is the entire target volume.

We detected the residual setup errors after correction of the patient’s position by use of Sentinel. PTV margins were all less than 5 mm after IGRT, which was decreased by 1~2 mm in all directions. The result was similar to the literature report: Batin *et al*.^21^ studied the residual errors detected by AlignRT in breast cancer after modified radical mastectomy. PTV margin were required to expand 3.5 mm, 2.4 mm, 4.0 mm in X, Y, and Z directions. Betgen *et al*.^23^ also studied the residual errors after correcting positions by AlignRT in breast cancer. They found that PTV margins were required to add 3.2 mm, 4.3 mm, 3.2 mm in three directions. In our center, PTV margins added were 5 mm in three shifting directions without considering rotation, and the PTV-boost margins were 7 mm, which are consistent with the experimental results by this study. After IGRT, PTV margins were all reduced to less than 5 mm. The residual errors are expected to be improved if a smaller slice thickness in the planning CT were used. Hence, in the future work to use Sentinel for assisting patient setup for every treatment session, we can expect to further decrease the margin of the PTV-boost, and that will result in reduced integral dose to patients without compromising the dose to the target. In addition, the staff of our department have strong sense of responsibility and tried their best during the process of fixation and setup of the patients to minimize the errors.

The advantages of Optical Laser 3D Surface imaging make it a certain value in clinical use, Batin *et al*.^21^ hold that surface imaging can offered more accurate positioning, shorter setup time and the reduction of imaging dose to patient from the traditional orthogonal X-ray setup technique. They have adopted this technique for all PBS PMRT treatment at their clinic. Studies by Padilla *et al*.^20^ have shown that instant feedback on patient position changes is a key benefit of surface imaging, which make this technique better for clinical use. Palotta *et al*.^24^ found that in patients with thoracic targets, the use of surface imaging resulted in improved positioning in 50%. Walter *et al*.^18^ reported the clinical data on the accuracy of patient positioning using the Catalyst system, suggesting that optical surface scanning by the Catalyst can be used for daily positioning at least to complement complementing conventional imaging modalities.

## Materials and Methods

This was a prospective study had approved from the Medical Ethics Committee of the Third Affiliated Hospital of Soochow University, and written informed consent was obtained from the patients before treatment. The methods used in this study were carried out in accordance with the guidelines outlined in the Declaration of Helsinki. This study enrolled 27 female patients with breast cancer who underwent radiotherapy after BCS from November 1, 2016 to April 30, 2017. The median age of the patients was 50 years old (range 35~64 years). The pathologic types of breast cancer are classified into non invasive carcinoma and invasive carcinoma, among which non invasive carcinoma includes ductal carcinoma *in situ* (DCIS) and Paget disease of the nipple not associated with invasive carcinoma and/or carcinoma *in situ* (DCIS) in the underlying breast parenchyma; in TNM staging, “Tis” represents non invasive carcinoma, on the other hand, T1 (Tumor ≤20 mm in greatest dimension) and T2 (Tumorå 20 mm but ≤50 mm in greatest dimension) represent invasive carcinoma^25^. The case number for intraductal carcinoma and invasive carcinoma of the study population were 6 and 21, the case numbers for T_is_, T_1_, and T_2_ stage were 6, 17, and 4, respectively; the N and M stage for all cases were N_0_ and M_0_. The characteristics of the study population are shown in Table 4.

**Table 4:** Characteristics of the study population (n = 27).

Clinical characteristics	Value
**The pathologic types**	
Intraductal carcinoma (n)	6
Invasive carcinoma (n)	21
**The pathological staging**	
pTisN0M0 (n)	6
pT1N0M0 (n)	17
pT2N0M0 (n)	4
Mammary gland volume (cm^3^)	479.4 ± 180.9
Age (years old)	49.5 ± 7.2
The average BMI (kg/m^2^)	24.8 ± 3.1

BMI: body mass index.

Patients were immobilized using a breast board (Posirest-2 Arm Support, CIVCO, US) in supine position with arms raised above the head. Planning CT scan was performed on a Sensation Open CT scanner (Siemens, Germany). CT images with a slice thickness of 5 mm covered the region from the annular membrane to the lower boarder of liver. Three longitudinal laser lines and one transversal laser line were marked on patient’s skin surface, and their intersecting points were tattoo marked for setup positioning at treatment.

For treatment planning, the clinical target volume (CTV) was delineated to include all breast tissues. The planning target volume (PTV) was defined as the CTV with a 5 mm margin, modified to exclude the volume adjacent to the skin surface within 5 mm. The CTV-boost volume consisted of the tumor bed, as marked by the surgery metal clips, postoperative serum swelling and surgical scars. The PTV-boost was defined as adding 7 mm margin to the CTV-boost and modified to be inside the PTV. Organ as risk (OAR) delineated were the lung, heart, and contralateral breast.

Dose prescription was 57.5 Gy in 25 fractions for PTV-boost and 47.5 Gy in 25 fractions for PTV. The dosimetric objectives were to cover 95% of the target volumes with the prescription dose and limit the OAR dose such that the V_5_ of ipsilateral lung was less than 50%, the mean dose to the heart less than 6 Gy, and the maximum dose of contralateral breast less than 5 Gy.

Treatment plans were generated using the Monaco treatment planning system (TPS) (Monaco 3.3, Elekta, Sweden). A single 190° arc was used for the beam setup. A 6MV photon beam was used. For the left-side treatment the arc started at gantry angle of 300° and ended at 130°, and for the right-side treatment the gantry angles were from 60° to 230°. The maximum number of control points was 150.

**Figure 2:**
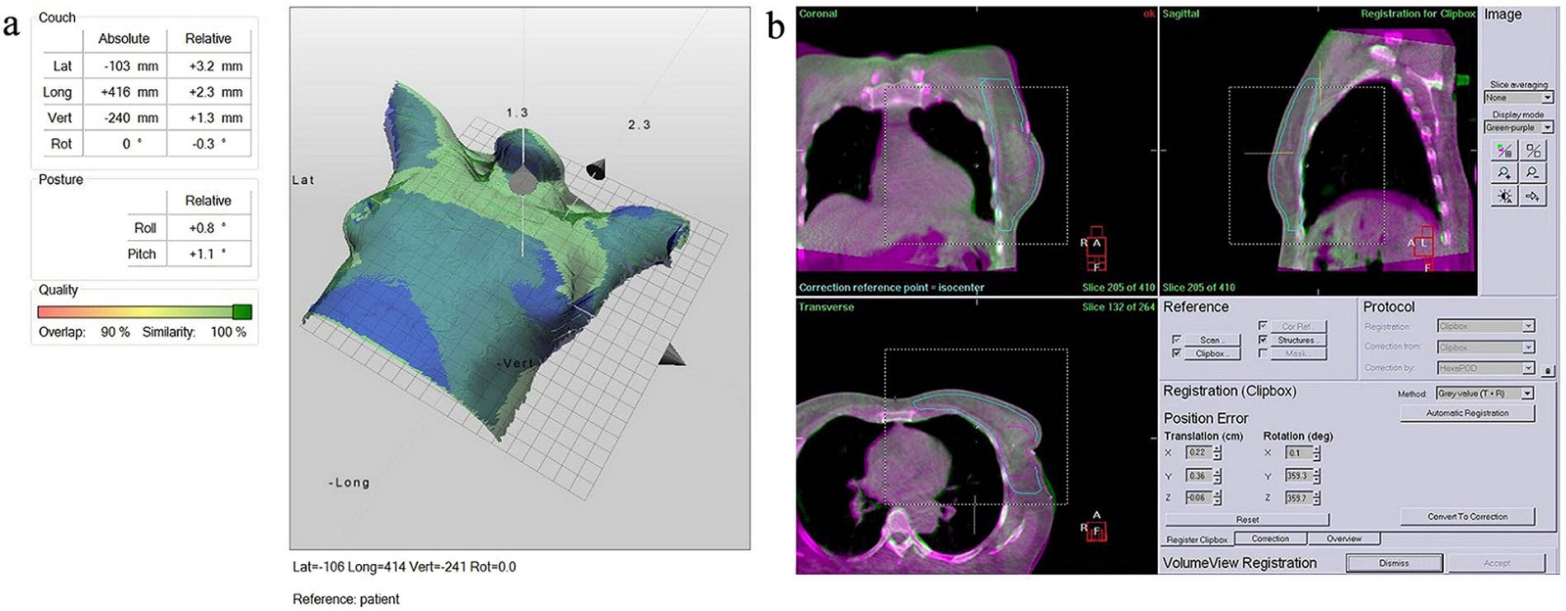
The images and data of setup corrections by Sentinel (**a**) and CBCT (**a**,**b**): The green area represents the body surface profile reconstructed by CT scan, the blue area represents the body surface profile reconstructed by Sentinel scan, and the dark blue represents areas that match with errors. The data on the left is the result of matching error.

**Figure 3:**
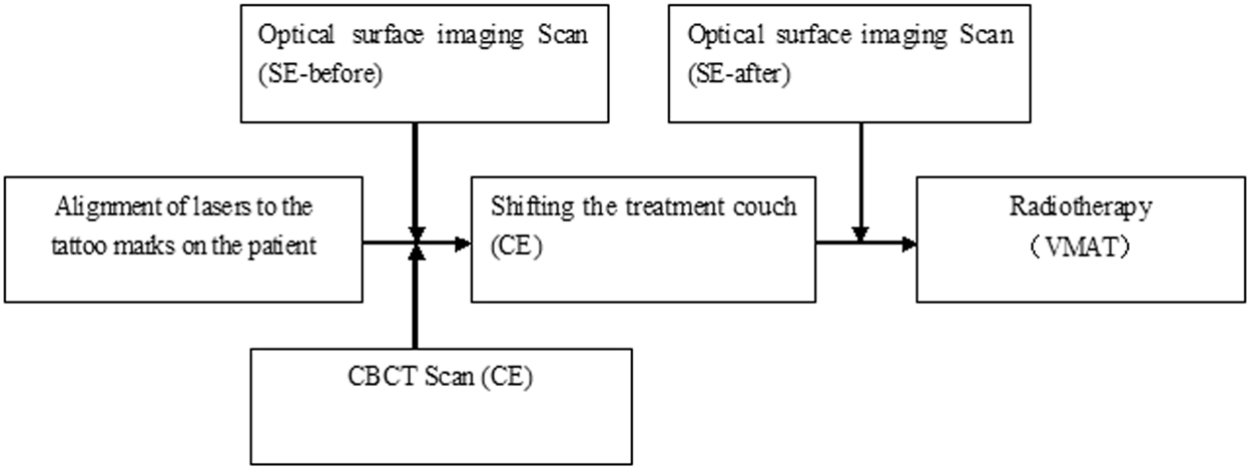
Flow chart of research steps.

This study was designed for evaluating the patient setup accuracy of using the optical surface imaging in the clinical setting but without deviating from the current CBCT based method for patient positioning correction. The starting point of patient setup was the alignment of treatment room lasers to the tattoo marks on the patient. Patient skin surface was acquired using the Sentinel system (C-RAD, Sweden), and the region of interest (ROI) consisted of the disease side breast and the adjacent chest wall. The upper boundary was the clavicle, and the medial boundary was the midline. The contralateral breast, chin, arm and armpit were excluded. The matching of the acquired surface ROI with the surface reconstructed by the planning CT yielded setup corrections that can be applied by a 6-dimensional (6D) treatment couch (HexaPOD, Elekta, Sweden). The shifts in the X, Y, Z directions and the three rotations about the X, Y, Z axes were recorded as a data set named SE-before (Sentinel errors before the treatment couch correction). Next, the routine CBCT scan was acquired for setup correction (XVI, Elekta, Sweden) using the Chest M20 mode, 120 kV, and F1 Bowtie filter. The ROI for matching the CBCT and planning CT included ipsilateral breast, chest wall, sternum and parts of the lung. Automatic registration was applied first followed by manual adjustment to best match the metal clips and the shape of the breast. The resulted couch movement by the 6D couch was obtained, denoted as CE (CBCT error), and the correction was applied using the automatic couch movement function for the HexaPOD couch. Prior to treatment delivery, the second optical surface imaging scan was performed, and the residual setup error as determined by the C-RAD software was denoted as SE-after (Sentinel error after shifting the treatment couch). The modified procedure to conduct the current study was facilitated by the MOSAIQ (Elekta, Sweden) Oncology Information System. In this workflow, it was the attending physician, Mengjiao Liu and Wendong Gu, and the technician, Xiaobo Wei, analysed the images and data for the first time, and then finished by the same technician, Xiaobo Wei. The images and data of setup corrections by Sentinel and CBCT is shown in Fig. 2, and a step by step procedure is shown in Fig. 3.

As the study was limited to assessment of patient setup accuracy with the optical surface imaging, comparison of initial couch setup errors between surface matching and CBCT matching with the planning CT was made. The correlation between the SE-before and CE was analyzed by Pearson correlation analysis using the SPSS 18.0 software. A correlation coefficient (r) ≤ 0.3 was considered with poor correlation, in the range 0.3~0.5 as moderately correlated, 0.5~0.8 as significantly correlated, equal to or greater than 0.8 as highly correlated.

The setup errors measured by SE-before and CBCT for the patient cohort can be used to determine the PTV margins required for treatment without couch movement correction. This is relevant to the margins for the PTV-boost in simultaneous integrated boost radiotherapy and PTV margins for partial breast radiotherapy. The PTV margins derived from SE-before and CBCT data were compared based on a statistical model. The average setup error and standard deviation (SD) of each patient was calculated for multiple treatment sessions. The group mean (M) of the study cohort was the average of the averaged error for each patient. The systematic error (Σ) was the standard deviation of the individual mean for each patient, and the randomize error (δ) was the root mean square of the mean square deviation of each patient^26^. The PTV margins were calculated by 2Σ + 0.7δ, based on the work by Sroom *et al*.^16^. For this study the SE-after was considered the residual error following CBCT based setup correction, and the resulting PTV margins were evaluated.

## Conclusion

In summary, optical surface imaging by Sentinel has a significant correlation with CBCT in detecting setup errors in postoperative radiotherapy for breast cancer. The differences of Σ and δ between the two IGRT methods were less than 1 mm. Sentinel can be used to replace CBCT to avoid unnecessary imaging radiation to patients.
